# The Anti‐Inflammatory Effect of Yangyin Tongnao Granule on Cerebral Ischemia–Reperfusion Injury in Rats

**DOI:** 10.1002/cns.70923

**Published:** 2026-05-11

**Authors:** Yangyang Zhang, Jiayang Wan, Tianxue Chen, Qianqian Chen, Jiehong Yang, Yu He, Huifen Zhou, Haitong Wan

**Affiliations:** ^1^ School of Pharmaceutical Sciences Zhejiang Chinese Medical University Hangzhou China; ^2^ College of Chinese Medicine for Cardiovascular‐Cranial Disease Zhejiang Chinese Medical University Hangzhou China; ^3^ Zhejiang Key Laboratory of Chinese Medicine for Cardiovascular and Cerebrovascular Disease Hangzhou China; ^4^ The First Affiliated Hospital Zhejiang University School of Medicine Hangzhou China; ^5^ School of Basic Medical Sciences Zhejiang Chinese Medical University Hangzhou China

**Keywords:** anti‐inflammatory, cerebral ischemia–reperfusion injury, JAK2/STAT3 signaling pathway, Yangyin Tongnao granule

## Abstract

**Background:**

Yangyin Tongnao (YYTN) granule is a classic traditional Chinese medicine (TCM) preparation known for its therapeutic efficacy in promoting blood circulation, resolving stasis, nourishing yin, and reinforcing qi. Its neuroprotective effects have been validated through both clinical and preclinical studies.

**Methods:**

A middle cerebral artery occlusion (MCAO) rat model was established, and rats were randomly assigned to five groups: Sham, MCAO, YYTN, Ag490 (JAK2 inhibitor), and Ag490 + YYTN. YYTN was administered intragastrically at 1.73 g/kg/day, while Ag490 was administered via lateral ventricle injection at a volume of 10 μL per rat. Neurological function was assessed using Garcia scores, and infarct volume was determined by TTC staining. Neuronal integrity and damage were evaluated through HE and Nissl staining. Plasma levels of IL‐18 and TNF‐α were measured via ELISA, and the mRNA expression of *IL‐6* and *IL‐1β* was quantified by RT‐qPCR. Inflammatory markers, including p‐JAK2, p‐STAT3, NLRP3, ASC, cl‐Caspase‐1, and cl‐IL‐1β, were assessed by Western blotting. The distribution of p‐JAK2 and p‐STAT3 in brain sections was visualized via immunofluorescence staining.

**Results:**

Treatment with YYTN significantly improved neurological function, reduced infarct volumes, and mitigated histopathological damage in the brain. Furthermore, YYTN significantly decreased plasma levels of IL‐18 and TNF‐α. In brain tissue, YYTN downregulated the mRNA expression of *IL‐6* and *IL‐1β* and reduced key inflammatory proteins, including p‐JAK2, p‐STAT3, NLRP3, ASC, cl‐Caspase‐1, and cl‐IL‐1β.

**Conclusion:**

These findings suggest that YYTN mitigates cerebral ischemia–reperfusion injury (CIRI)‐induced neuroinflammation by inhibiting the JAK2/STAT3 signaling pathway.

## Graphical abstract

This study shows that Yangyin Tongnao (YYTN) granule alleviates brain damage and functional deficits in MCAO rats. Mechanistically, YYTN inhibits the activation of the JAK2/STAT3 signaling pathway and downstream NLRP3 inflammasome and reduces both systemic (plasma IL‐18, TNF‐α) and local cerebral (*IL‐6*, *IL‐1β* mRNA) inflammatory markers.

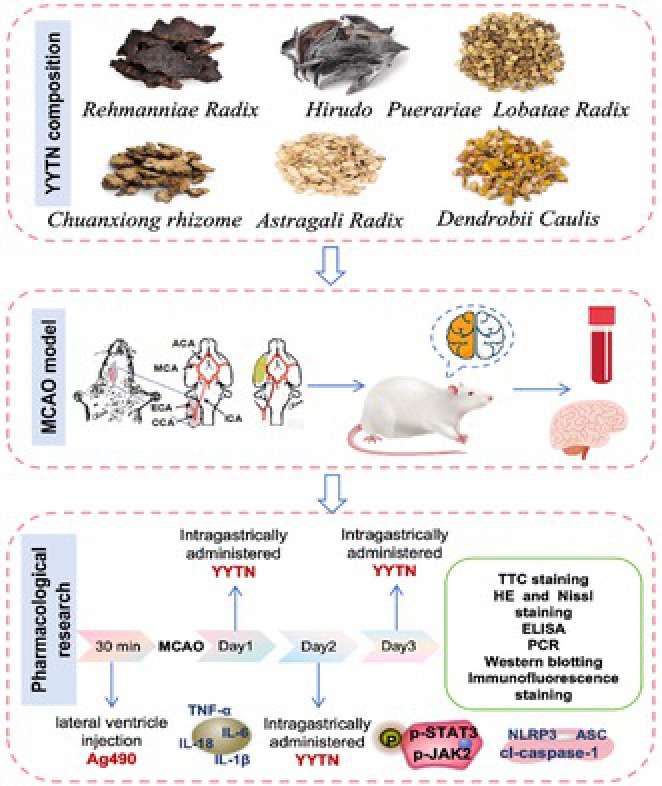

**Abbreviations**
CCA: common carotid artery
CIRI: cerebral ischemia–reperfusion injury
ECA: external carotid artery
ELISA: enzyme‐linked immunosorbent assay
HE: hematoxylin and eosin
ICA: internal carotid artery
IL: interleukin
JAK: janus kinase
MCAO: middle cerebral artery occlusion
NLRP: nucleotide‐binding oligomerization domain‐like receptor protein
RT‐qPCR: reverse transcription‐quantitative polymerase chain reaction
SD: Sprague Dawley
SPF: specific pathogen‐free
STAT: signal transducers and activators of transcription
TCM: traditional Chinese medicine
TNF: tumor necrosis factor
tPA: tissue plasminogen activator
TTC: 2, 3, 5‐triphenyltetrazolium chloride
YYTN: Yangyin Tongnao

## 1 Introduction

Cardiovascular and cerebrovascular disorders contribute markedly to global mortality and morbidity, especially among the elderly population. The incidence of these conditions continues to rise, presenting a significant public health burden. Cerebrovascular disease refers to a disease in which cerebral blood flow disorder or abnormal vascular structure causes massive hemorrhage or insufficient blood supply to the brain [1]. Ischemic stroke is characterized by insufficient cerebral blood supply due to vascular occlusion and accounts for the majority of these cases. Alarmingly, the global incidence of ischemic stroke has nearly doubled over the past two decades [2]. Despite major advances in medical science and healthcare infrastructure, there remain few options to treat ischemic stroke due to the complex pathophysiology of this disease.

Tissue plasminogen activator (tPA) is the only agent with National Drug Administration approval for acute ischemic stroke [3]. Traditional Chinese medicine (TCM), with its holistic approach and the principle of syndrome differentiation, offers unique therapeutic advantages for complex diseases such as ischemic stroke. TCM is characterized by its multi‐component, multi‐target, and multi‐pathway mechanisms, which contribute to its efficacy in managing cerebrovascular conditions [4, 5]. Yangyin Tongnao (YYTN) granule is a TCM formulation that consists of *Conioselinum anthriscoides ‘Chuanxiong’* [Apiaceae], *Astragalus mongholicus* Bunge [Fabaceae], *Pueraria montana var. lobata* (Willd.) Maesen & S.M. Almeida ex Sanjappa & Predeep [Fabaceae], *Dendrobium nobile* Lindl. [Orchidaceae], and *Rehmannia glutinosa* (Gaertn.) DC. [Orobanchaceae]. The neuroprotective effects of YYTN have been validated through both clinical and preclinical studies [6]. However, the mechanistic basis for the therapeutic effects of YYTN on cerebral ischemia–reperfusion injury (CIRI) is not clear.

Recent research on cerebral ischemic injury has focused on neuroprotective mechanisms such as anti‐apoptosis, inhibition of oxidative stress, and modulation of the inflammatory response [7, 8]. During reperfusion following ischemia, the ischemic core becomes a focal point for the release of numerous inflammatory mediators, triggering a cascade of immune and inflammatory responses [9]. Among the pathways implicated in this process, JAK/STAT is crucial for mediating inflammation [10]. Phosphorylated STATs undergo nuclear translocation and bind particular sequences of the DNA to control gene expression. Another major contributor to inflammation is the NLRP3 inflammasome, a multi‐protein complex composed of the NLRP3 receptor, apoptosis‐associated speck‐like protein containing a caspase activation and recruitment domain (ASC), and pro‐caspase‐1 [11, 12]. Upon activation during cerebral ischemia, caspase‐1 cleaves pro‐inflammatory cytokines into their active forms, amplifying the inflammatory response [13].

Here, the effects of YYTN on inflammation in a rat model of CIRI were investigated, with a focus on JAK2/STAT3 signaling and downstream inflammasome activation.

## 2 Materials and Methods

### 2.1 Animals

Sprague Dawley (SD) rats (male, 280–300 g) with specific pathogen‐free (SPF) grade were supplied and maintained by the Zhejiang Chinese Medical University Laboratory Animal Research Center. The animals were kept in a controlled environment (12 h light/dark cycle, 22°C ± 3°C, 60% ± 5% relative humidity) with unrestricted water and food for a 7‐day acclimation period. Prior to surgery, animals were fasted for 12 h but were allowed water.

### 2.2 Establishment of Middle Cerebral Artery Occlusion (MCAO) Model

Focal cerebral ischemia was induced using a modified version of the Zea Longa method [14]. Anesthesia was administered via intraperitoneal injection of Zoletil 50 (40 mg/kg), preceded by an intramuscular injection of atropine (0.04 mg/kg) given 5 min earlier to reduce respiratory secretions. Following the onset of anesthesia, a midline cervical incision was used to achieve right common carotid artery (CCA), internal carotid artery (ICA), and external carotid artery (ECA) exposure. A 4–0 nylon monofilament (0.26 mm diameter), coated with polylysine, was carefully introduced into the ICA through the CCA to a depth of 18–20 mm to occlude the origin of the middle cerebral artery. The filament was carefully removed after 1 h to enable reperfusion, and the incision was closed with sutures. The success of the MCAO model was independently verified using the following methods: (1) Neurological function was evaluated 24 h after cerebral ischemia–reperfusion using the Longa neurological score, and rats with a neurological deficit score ≥ 1 were considered successfully modeled; (2) At the end of the experiment, TTC staining was performed to confirm the presence and consistency of infarct volume in the model group. Animals were excluded if they met any of the following criteria: (1) No neurological deficit symptoms 24 h after reperfusion (Longa score < 1); (2) Subarachnoid hemorrhage observed by visual inspection during brain extraction; (3) Rats died 24 h after reperfusion.

### 2.3 Experimental Groups and Drug Treatment

Following successful MCAO induction and strict exclusion according to the predefined criteria, a total of 90 rats were successfully modeled and included in the final analysis. Rats were randomly assigned to five groups: Sham, MCAO, YYTN, Ag490 (JAK2 inhibitor), and Ag490 + YYTN groups, with 18 rats in each group. Rats in the YYTN group were intragastrically administered YYTN at a dose of 1.73 g/kg/day, starting on the day of surgery and continuing once daily for 3 consecutive days. The drug dosage was based on a conversion from the clinical human dosage. The clinical dose of YYTN was 16.5 g/day, and the rat dose was calculated using the formula: (6.3 × 16.5 g/day)/60 kg = 1.73 g/kg/day. In the Ag490 and combination groups, Ag490 (10 mM) was administered via lateral ventricle injection at a volume of 10 μL per rat. The stereotaxic coordinates referenced to bregma are mediolateral (ML) = 1.2 mm, anteroposterior (AP) = −0.8 mm, and dorsoventral (DV) = −4.0 mm. The Sham and MCAO groups received an equal volume of normal saline corresponding to YYTN via oral gavage and an equal volume of normal saline corresponding to Ag490 via lateral ventricle injection at the same respective schedule.

### 2.4 TTC Staining

The brain tissues were kept at −20°C for 15 min. The frozen brains were then sliced into coronal sections and incubated in a 2% TTC solution at 37°C away from light. Following incubation, the slices were carefully removed and imaged. The infarct volume was quantified using ImageJ.

### 2.5 Neurological Functional Assessment

Neurological deficits were evaluated using the Garcia scores [15], which assess both motor and sensory function across six domains: Spontaneous activity (0–3 points), symmetry of limb movement (0–3 points), forelimb extension (0–3 points), ability to climb a wire cage (1–3 points), response to tactile stimulation on both sides of the body (1–3 points), and response to tactile vibration (1–3 points). The maximum possible score of 18 points indicates no neurological impairment, whereas the minimum score of 3 reflects severe neurological dysfunction.

### 2.6 Tissue Staining

Paraffin‐embedded brain tissue sections underwent graded ethanol dehydration, rinsing in distilled water, and staining with hematoxylin and eosin (H&E) before clearing with xylene and mounting with neutral resin. Histopathological changes were observed under a light microscope, and relevant regions were analyzed. For Nissl staining, brain sections were incubated in Nissl stain solution at 60°C for 20 min. After triple rinsing in distilled water, the sections were dried at 60°C, cleared with xylene, and sealed with neutral gum. Neuronal integrity and damage were assessed microscopically based on Nissl body morphology.

### 2.7 Enzyme‐Linked Immunosorbent Assay **(ELISAs)**

Levels of inflammatory cytokines were assessed using ELISA kits as directed. Samples and reagents were added to 96‐well microplates, and absorbances at 450 nm were measured with a microplate reader.

### 2.8 Reverse Transcription‐Quantitative Polymerase Chain Reaction **(RT‐qPCR)**

Total RNA was isolated from brain tissues using TRIzol. cDNA synthesis was conducted with the 5× All‐In‐One RT MasterMix kit. Quantitative PCR was undertaken using the Blastaq 2× qPCR MasterMix kit. Reaction conditions: 95°C, 10 min denaturation; 95°C, 15 s; 60°C, 60 s; 40 cycles. Primer sequences are provided in Table 1.

**TABLE 1:** Primer sequences.

Gene	Primers	Sequences\
*IL‐6*	Forward	5′‐AGTTCCGTTTCTACCTG‐3′
	Reverse	5′‐GAATGACTCTGGCTTTG‐3′
*IL‐1β*	Forward	5′‐GACTTCACCATGGAACCCGT‐3′
	Reverse	5′‐GGAGACTGCCCATTCTCGAC‐3′
*GAPDH*	Forward	5′‐GCTGAGAATGGGAAGCTGGT‐3′
	Reverse	5′‐GGTGGTGAAGACGCCAGTAG‐3′

### 2.9 Western Blotting

Total proteins were isolated from brain tissue lysates prepared using cold lysis buffer. BCA assays were used for protein quantification. Proteins were resolved via SDS‐PAGE and transferred to PVDF membranes, which were probed with primary antibodies against JAK2, phospho‐JAK2 (p‐JAK2), STAT3, phospho‐STAT3 (p‐STAT3), NLRP3, ASC, cleaved Caspase‐1 (cl‐Caspase‐1), and cleaved IL‐1β (cl‐IL‐1β). GAPDH was used as the internal loading control.

### 2.10 Immunofluorescence Staining

Immunofluorescence was employed to visualize the distribution of p‐JAK2 and p‐STAT3 in brain sections. After incubation with primary antibodies at 4°C overnight, sections were washed with PBS and treated with fluorescently labeled secondary antibodies for 1 h before sealing with glycerol and imaging under the fluorescence microscope.

### 2.11 Statistical Analysis

Data were analyzed with SPSS 25.0. One‐way analyses of variance (ANOVAs) were used for comparisons among multiple groups. For skewed data, the Kruskal–Wallis H test was used. All data were expressed as mean ± standard deviation ($\bar{x}$±s). *p* < 0.05 was considered statistically significant.

## 3 Results

### 3.1 YYTN Reduces Infarct Volume in MCAO Rats

**FIGURE 1:**
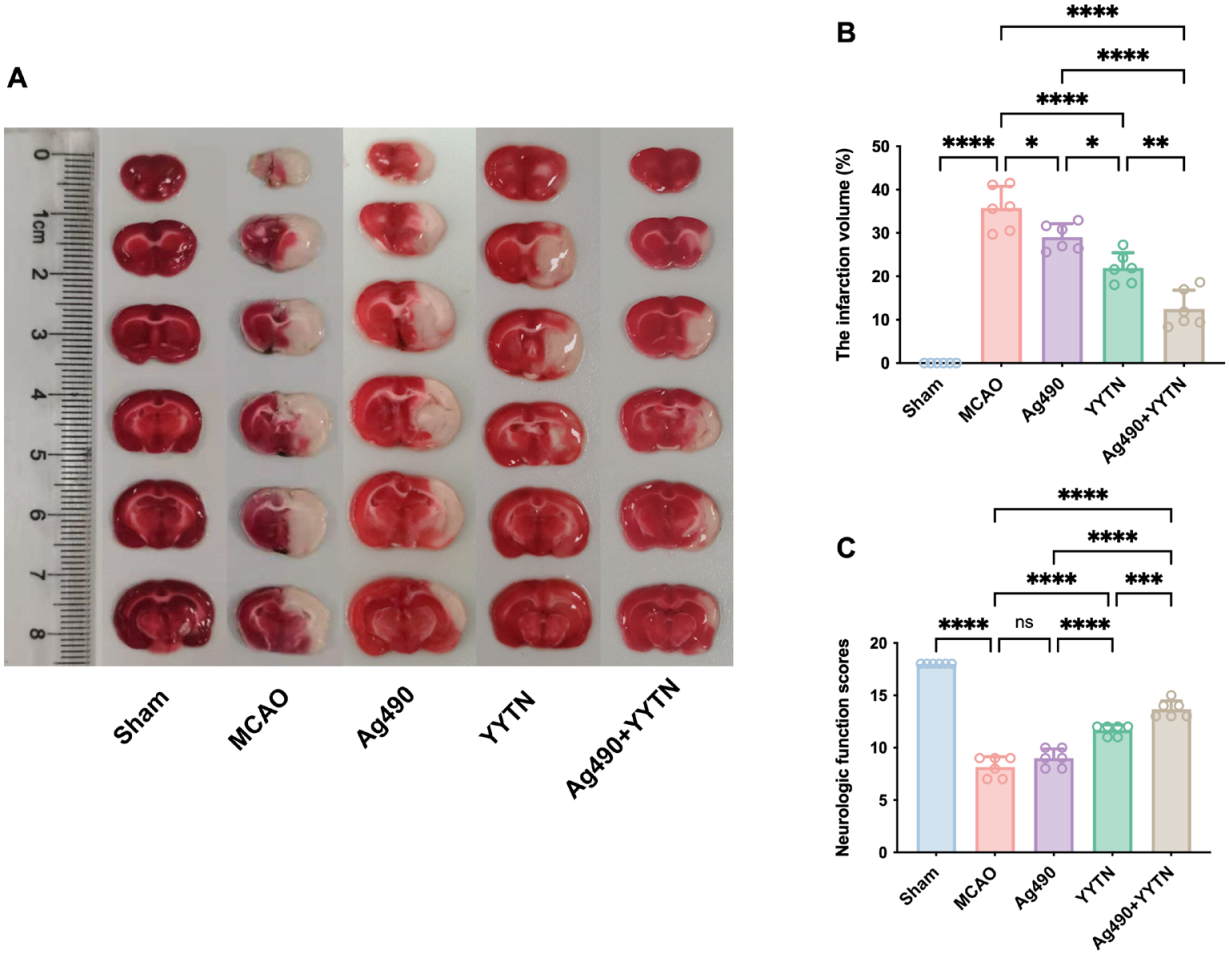
The representative pictures of TTC staining (A), the infarction volume (B), and the neurological function scores (C) of rats ($\bar{x}$±s, *n* = 6). ns *p* > 0.05, *p < 0.05, **p < 0.01, ***p < 0.001, ****p < 0.0001.

Representative TTC‐stained brain sections are shown in Figure 1A, and infarct volume quantification is presented in Figure 1B. No infarction was observed in the Sham group. The MCAO group displayed markedly greater infarct volumes (*p* < 0.0001). In contrast, infarct volume was significantly reduced in the Ag490 group (*p* < 0.05) and more markedly decreased in the YYTN group (*p* < 0.0001). The combination treatment group (Ag490 + YYTN) exhibited the most pronounced reduction, with infarct volumes significantly reduced compared to the Ag490 group (*p* < 0.0001).

### 3.2 YYTN Improves Neurological Function in MCAO Rats

The Garcia score was utilized to score neurological function (Figure 1C). Rats in the Sham group exhibited normal neurological behavior with an average score of 18, indicating no deficits. In contrast, MCAO rats showed significantly lower scores (*p* < 0.0001), reflecting marked neurological impairment. Treatment with YYTN or YYTN combined with Ag490 markedly improved the scores relative to the MCAO group (*p* < 0.0001). Additionally, scores in the Ag490 + YYTN group were markedly greater than those in rats treated only with Ag490 (*p* < 0.0001).

### 3.3 YYTN Mitigates Neuronal Damage in MCAO Rats

**FIGURE 2:**
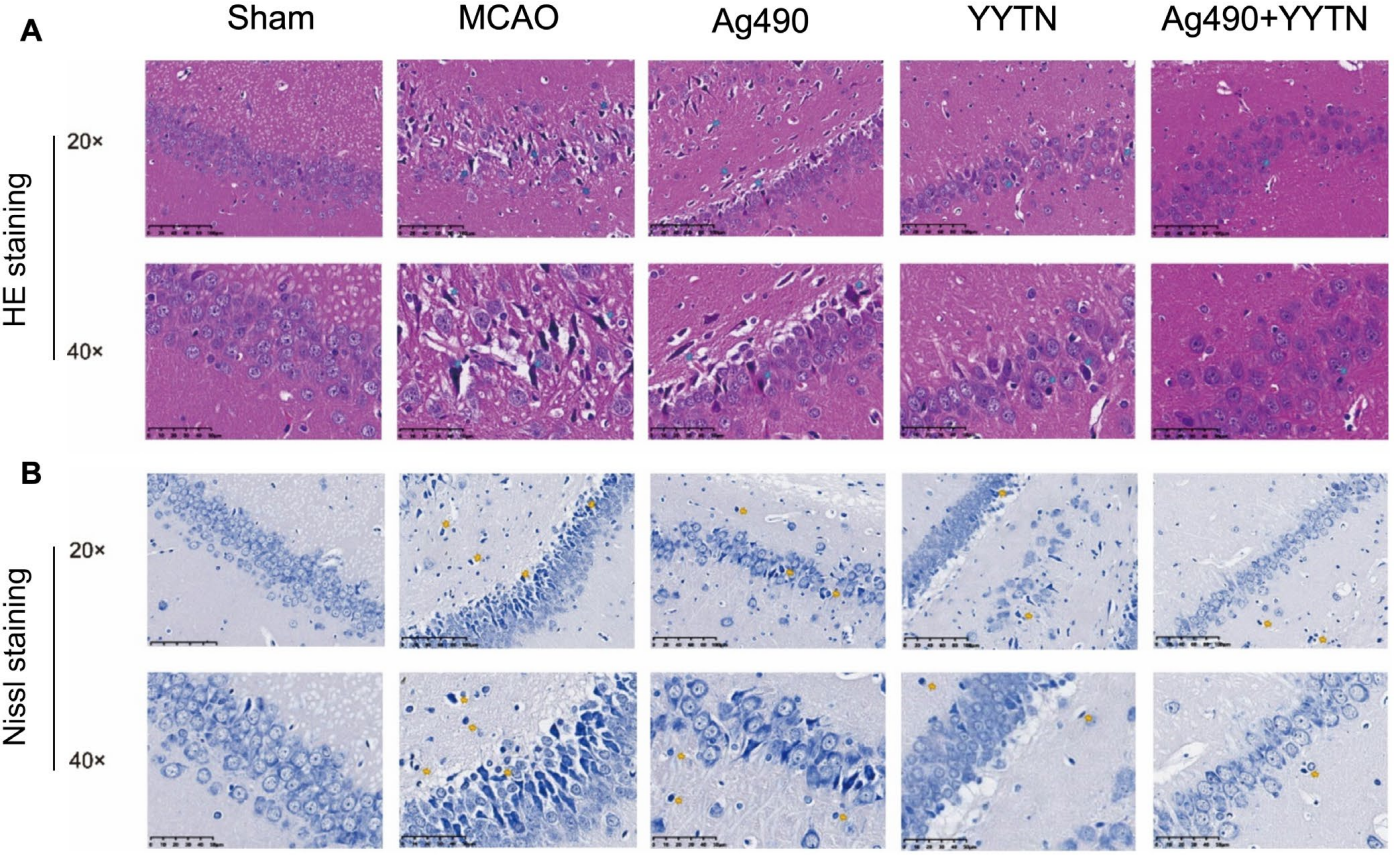
The HE staining (A) and the Nissl staining (B) of brain tissue in rats. Blue arrows point to inflammatory cells; Yellow arrows point to condensed cells.

Histological analysis of HE‐stained sections (Figure 2A) revealed that brain tissue from Sham rats showed well‐preserved cellular architecture, including intact, neatly arranged neurons with lightly stained nuclei and prominent nucleoli. In contrast, the MCAO group exhibited disorganized tissue structure, nuclear condensation or disappearance, and large vacuolated regions. The Ag490 group showed disorganized cell structures similar to the MCAO group. However, YYTN treatment restored tissue integrity and reduced neuronal damage. The Ag490 + YYTN group displayed histological features closely resembling those of the Sham group, indicating substantial neuroprotection.

### 3.4 YYTN Attenuates Nissl Body Loss in MCAO Rats

As illustrated in Figure 2B, the Nissl‐stained brain sections from the Sham group showed dense and well‐defined neurons with clearly visible nuclei and nucleoli stained dark blue. In the MCAO group, neuronal density was reduced, with evidence of cell shrinkage and dissolution of Nissl bodies, indicating severe damage. The Ag490 group exhibited slight improvements in cell arrangement but no significant changes in Nissl body integrity. YYTN treatment mitigated neuronal loss and reduced pyknosis, while the Ag490 + YYTN group showed considerable preservation of neuronal morphology, closely approximating the Sham condition.

### 3.5 YYTN Attenuates Inflammatory Cytokine Production in MCAO Rats

**FIGURE 3:**
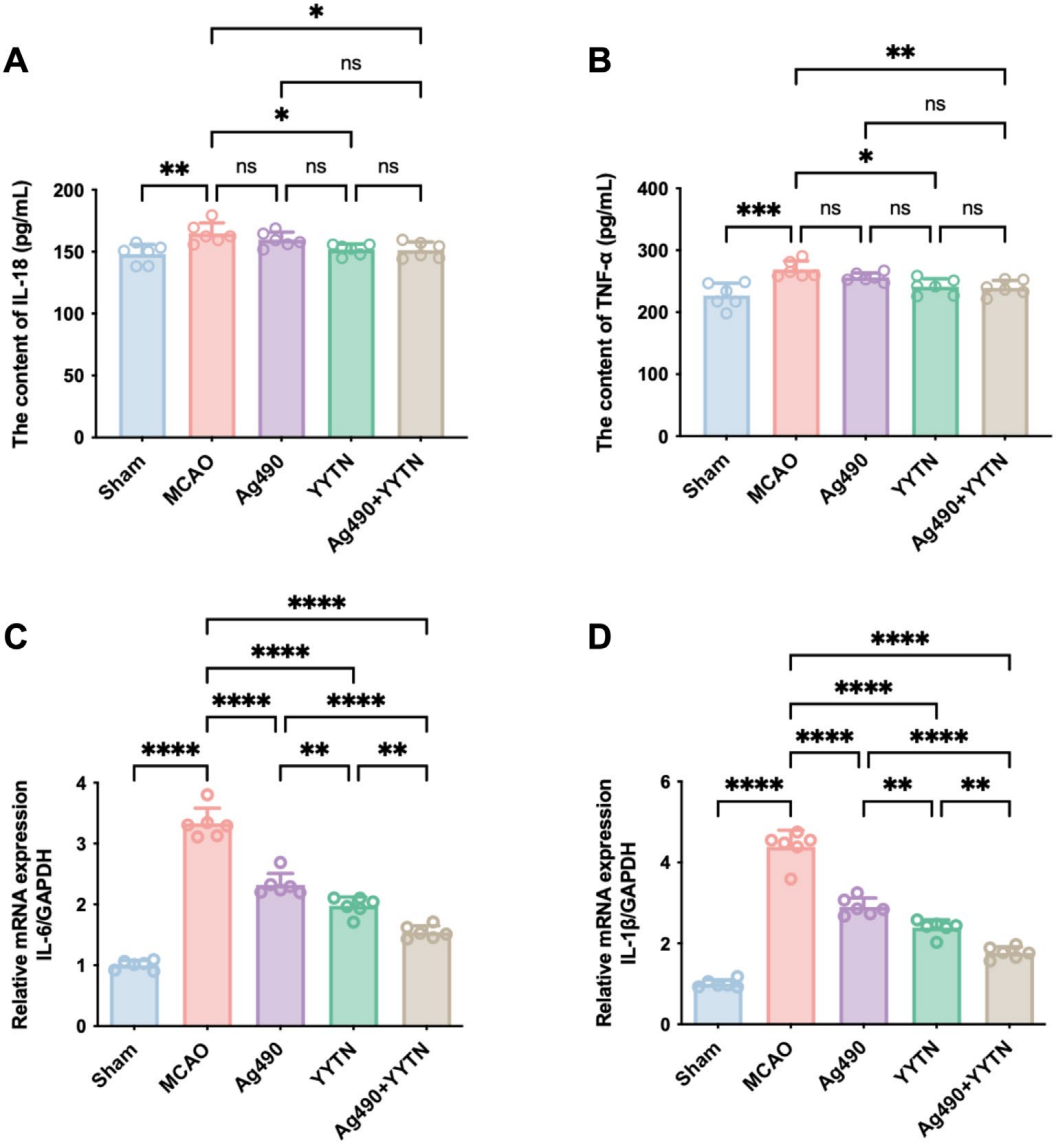
The contents of inflammatory factors of IL‐18 (A) and TNF‐α (B) of plasma, and the expression of *IL‐6* (C) and *IL‐1β* (D) mRNA of brain tissue in rats ($\bar{x}$±s, *n* = 6). Ns *p* > 0.05, *p < 0.05, **p < 0.01, ***p < 0.001, ****p < 0.0001.

As shown in Figure 3A,B, plasma IL‐18 and TNF‐α contents were markedly raised in the MCAO group relative to the Sham group (*p* < 0.01 or *p* < 0.001), indicating a robust systemic inflammatory response following ischemic injury. Treatment with YYTN and Ag490 + YYTN significantly decreased these elevated cytokine levels in plasma (*p* < 0.05 or *p* < 0.01), suggesting a potential synergistic anti‐inflammatory effect.

### 3.6 YYTN Decreases *IL‐6* and *IL‐1β* mRNA Expression in MCAO Rats

As shown in Figure 3C,D, cerebral ischemia–reperfusion induced a marked increase in the brain *IL‐6* and *IL‐1β* mRNA levels in MCAO model rats compared to the Sham group (*p* < 0.0001). Administration of Ag490, YYTN, or the combination of both significantly decreased the expression of these inflammatory genes relative to the MCAO group (*p* < 0.0001). Moreover, *IL‐6* and *IL‐1β* mRNA levels in the Ag490 + YYTN group were markedly reduced relative to animals treated with Ag490 alone (*p* < 0.0001).

### 3.7 YYTN Suppresses JAK2/STAT3 Pathway‐Related Protein Expression in MCAO Rats

**FIGURE 4:**
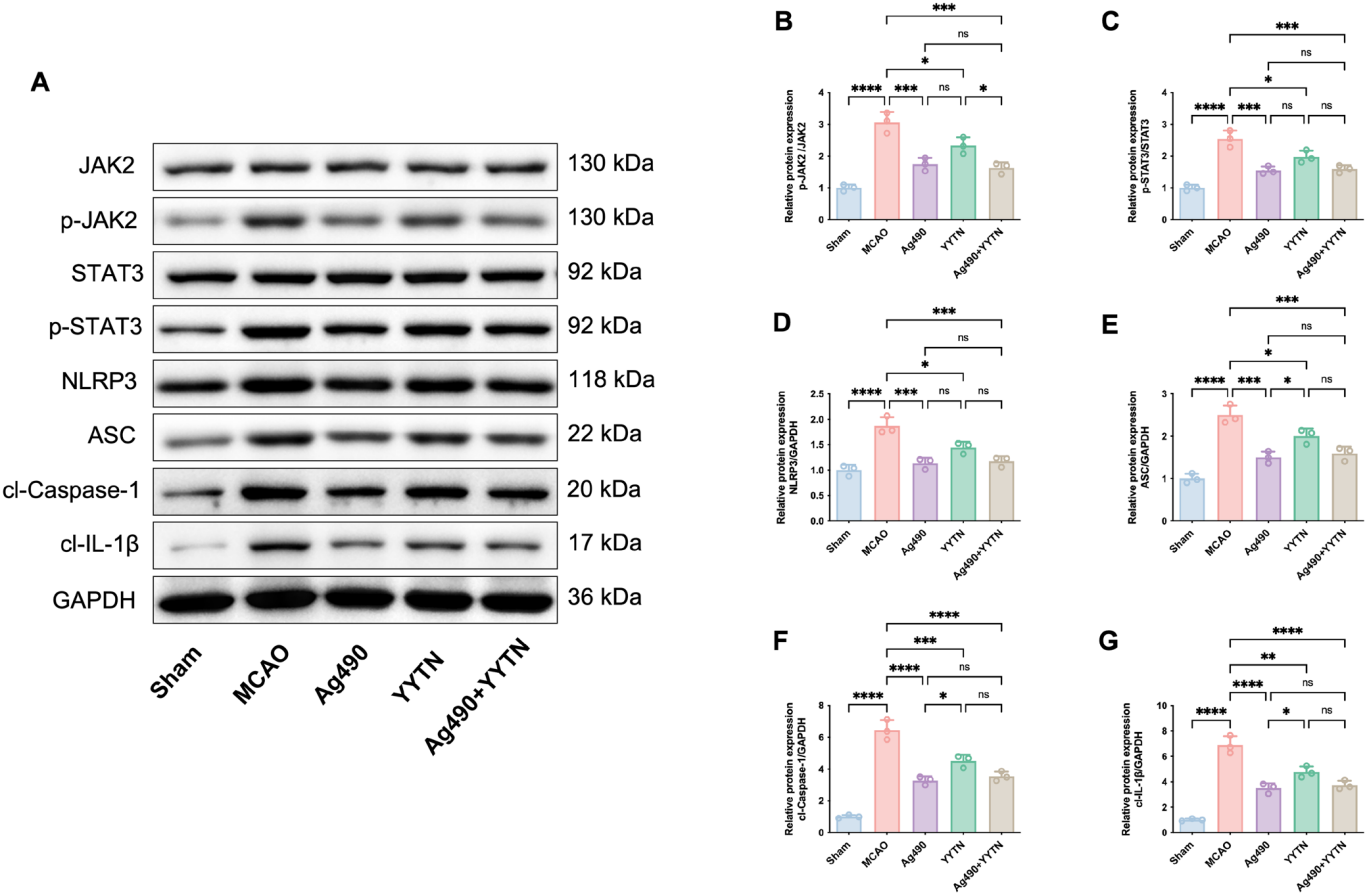
The representative pictures of Western blotting (A) and the protein expression (B, p‐JAK2; C, p‐STAT3; D, NLRP3; E, ASC; F, cl‐Caspase‐1; G, cl‐IL‐1β) of brain tissue in rats ($\bar{x}$±s, *n* = 3). ns *p* > 0.05, *p < 0.05, **p < 0.01, ***p < 0.001, ****p < 0.0001.

Figure 4A–G depicts the protein expression profiles of key mediators in the JAK2/STAT3 signaling axis and its downstream effectors. Compared to the Sham group, the MCAO group exhibited significantly elevated levels of p‐JAK2, p‐STAT3, NLRP3, ASC, cl‐Caspase‐1, and cl‐IL‐1β (*p* < 0.0001). Treatment with Ag490 led to a significant downregulation of these proteins (*p* < 0.001 or *p* < 0.0001), confirming its inhibitory action on the pathway. Similarly, YYTN administration significantly reduced their expression (*p* < 0.05, *p* < 0.01 or *p* < 0.001), though to a slightly lesser extent, suggesting partial modulation of this signaling cascade by the herbal compound.

### 3.8 YYTN Reduced p‐JAK2 and p‐STAT3 Expression in MCAO Rats

**FIGURE 5:**
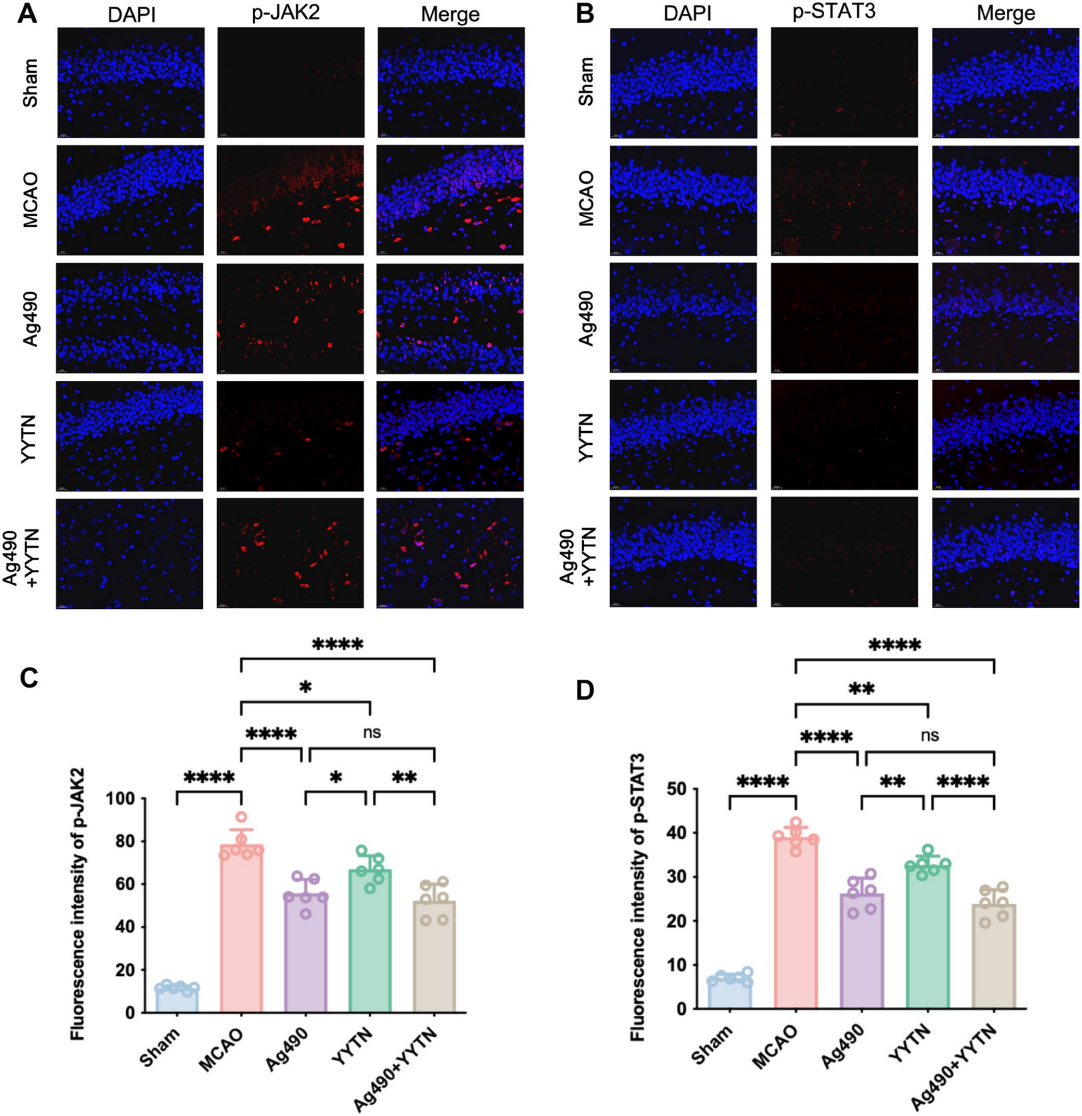
The representative pictures of immunofluorescence staining (A and B), and the fluorescence intensity of p‐JAK2 (C) and p‐STAT3 (D) of brain tissue in rats ($\bar{x}$±s, *n* = 6). ns *p* > 0.05, *p < 0.05, **p < 0.01, ****p < 0.0001.

The representative pictures of immunofluorescence staining were shown in Figure 5A,B, and the fluorescence intensity of p‐JAK2 and p‐STAT3 was shown in Figure 5C,D, respectively. The fluorescence intensities of both p‐JAK2 and p‐STAT3 were markedly increased in the MCAO model group compared to the Sham group (*p* < 0.0001), indicating activation of the JAK2/STAT3 pathway after ischemia–reperfusion. Compared with the MCAO group, treatment with Ag490 and YYTN significantly reduced the fluorescence intensities of both p‐JAK2 and p‐STAT3 (*p* < 0.05, *p* < 0.01 or *p* < 0.0001), and the combination of Ag490 and YYTN further suppressed the fluorescence intensities, showing a more pronounced effect than either treatment alone. These results suggest that YYTN can inhibit the activation of the JAK2/STAT3 pathway in CIRI.

## 4 Discussion

Cerebral ischemic diseases contribute significantly to global mortality and long‐term disability [16]. The pathophysiological consequences of CIRI, including secondary damage induced by oxidative stress and inflammation, exacerbate the overall injury burden. In this study, the protective effect of YYTN on CIRI and its molecular mechanism were detected by the MCAO rat model. The findings show that YYTN can not only significantly improve neurological deficits, reduce cerebral infarction volume, and reduce brain tissue pathological damage, but also effectively inhibit the inflammatory response at the systemic and local levels. The core mechanism may be related to inhibiting the activation of the JAK2/STAT3 signaling pathway, thereby down‐regulating the expression of downstream NLRP3 inflammasome and pro‐inflammatory factors.

TCM formulations, particularly those with multitarget mechanisms, have gained attention for their therapeutic efficacy in ischemic stroke. YYTN is a classic Chinese medicinal preparation known for its ability to promote blood circulation, resolve stasis, nourish yin, and reinforce qi, making it ideally suited for treating ischemic stroke [17]. In previous studies, the NADES extraction process of YYTN was optimized using a genetic neural network [18], and the spectrum‐effect relationship between HPLC fingerprints and antioxidant activity of YYTN was established using Pearson product–moment correlation coefficient (PPMCC) and multiple linear regression analysis (MLRA) [19]. Our previous studies have demonstrated its pronounced neuroprotective effects in cerebral ischemia–reperfusion models [20, 21]. Network pharmacology research and experimental verification indicate that YYTN can ameliorate CIRI in rats by regulating the HIF‐1α pathway [22]. Mechanistic studies have shown that YYTN can inhibit neuroinflammation through the Wnt signaling pathway and promote neural stem cell‐mediated brain repair [23]. Following cerebral ischemia, patients experience a series of adverse neurological functional changes [24], which coincide with cerebral infarction and pathological brain tissue changes. YYTN significantly alleviated the neurological deficits, reduced the infarct volume, and improved the cytoplasmic and nuclear abnormalities. This result is consistent with the effect of YYTN in the theory of TCM for its nourishing yin and activating blood, dredging collaterals and opening orifices, and improving cerebral circulation and nerve function, and it also provides an experimental basis for the clinical efficacy of YYTN in the treatment of cerebral ischemic diseases from the perspective of modern medicine.

YYTN plays a crucial role in the treatment of ischemic stroke through multiple targets and pathways, particularly in its neuroinflammatory protective effects. Our findings further reveal that YYTN significantly reduces the levels of IL‐18 and TNF‐α in plasma, suggesting that it has a systemic anti‐inflammatory effect. More importantly, YYTN down‐regulated the mRNA expression of key pro‐inflammatory cytokines (*IL‐6* and *IL‐1β*) in injured brain tissue, directly indicating its ability to target the injured lesion and inhibit local neuroinflammation at the gene transcription level. These results provide a solid foundation for understanding the potential of YYTN in alleviating the inflammatory cascade associated with ischemic stroke.

The In‐depth findings of this study revealed the key signaling pathway for YYTN to exert anti‐inflammatory effects. The JAK2/STAT3 pathway is a classic inflammatory signal transduction hub, which is rapidly activated after cerebral ischemia and drives the expression of a large number of inflammatory genes [25]. Ag490, a specific inhibitor of JAK2, was used as a reference compound. Our results showed that YYTN and Ag490 significantly reduced the levels of p‐JAK2 and p‐STAT3 proteins in brain tissue, indicating that they effectively blocked the activation of this pathway. YYTN also significantly reduced the expression of key components of the NLRP3 inflammasome (NLRP3, ASC) and its effector protein (cl‐Caspase‐1), and ultimately reduced its active product (cl‐IL‐1β). Existing studies have confirmed that the activation of the JAK2/STAT3 signaling pathway can directly upregulate NLRP3 and promote the assembly and activation of inflammasomes. Therefore, the study demonstrated that YYTN may inhibit the assembly and activation of the NLRP3 inflammasome by inhibiting JAK2/STAT3 phosphorylation and ultimately reducing caspase‐1‐dependent IL‐1β maturation and release. These findings provide critical insights into the multi‐target anti‐inflammatory mechanism of YYTN.

This study systematically explained the mechanism of YYTN against CIRI neuroinflammations and provided a new modern pharmacological basis for the treatment of ischemic stroke with TCM. However, there are still some limitations in this study. Firstly, as a TCM formulation, the specific active components of YYTN that play a key role are not yet clear. Secondly, although Ag490 was used as an inhibitor of JAK2, the upstream and downstream of the JAK2/STAT3 pathway and the NLRP3 inflammasome still need to be verified more directly by interventions such as gene knockout or inhibitors. Finally, this study was conducted using animal models, which may limit the direct translation of the findings to clinical settings. In addition, cerebral blood flow (CBF) was not monitored during surgery, which should be included in future studies to ensure more accurate evaluation of the MCAO model. Furthermore, complementary cellular analyses are needed to further elucidate the underlying mechanisms.

## 5 Conclusion

In summary, this study demonstrates that YYTN confers neuroprotection against CIRI by attenuating neuroinflammation and associated neuronal damage. Mechanistically, the therapeutic effect is linked to the inhibition of the JAK2/STAT3 signaling pathway. YYTN significantly reduced plasma inflammatory markers (IL‐18 and TNF‐α), while in brain tissue, it down‐regulated NLRP3 inflammasome components (NLRP3, caspase‐1, IL‐1β) and decreased pro‐inflammatory cytokines (*IL‐6* and *IL‐1β*). These findings not only elucidate a key pharmacological mechanism underlying the neuroprotective action of YYTN but also provide a scientific foundation for its clinical application in the prevention and treatment of ischemic cerebrovascular diseases within the framework of TCM.

## Author Contributions

Yangyang Zhang: Methodology, Visualization, Funding acquisition, and Writing – original draft; Jiayang Wan: Methodology, Validation, and Visualization; Tianxue Chen: Formal analysis; Qianqian Chen: Data curation; Jiehong Yang: Software; Yu He: Investigation and Supervision; Huifen Zhou: Writing – review and editing and Conceptualization; Haitong Wan: Funding acquisition and Supervision.

## Ethics Statement

The animal experiment was approved by the Experimental Animal Management and Ethics Committee of Zhejiang Chinese Medical University (approval no. IACUC‐20221114–04).

## Conflicts of Interest

The authors declare no conflicts of interest.

## Data Availability

The data that support the findings of this study are available from the corresponding author upon reasonable request.
